# Aminopeptidase O Protein mediates the association between *Lachnospiraceae* and appendicular lean mass

**DOI:** 10.3389/fmicb.2024.1325466

**Published:** 2024-02-07

**Authors:** Bingjun Gao, Zhonghua Zhou, Junfei Chen, Shengling Zhang, Shaobin Jin, Weiwei Yang, Yinghan Lei, Kunyao Wang, Jinxu Li, Yan Zhuang

**Affiliations:** Department of Pediatric Surgery, Qilu Hospital of Shandong University, Jinan, Shandong, China

**Keywords:** AOPEP, Mendelian randomization, gut microbiota, appendicular lean mass, sarcopenia

## Abstract

**Objective:**

Investigating the causal relationship between *Lachnospiraceae* and Appendicular lean mass (ALM) and identifying and quantifying the role of Aminopeptidase O Protein (AOPEP) as a potential mediator.

**Methods:**

The summary statistics data of gut microbiota composition from the largest available genome-wide association study (GWAS) meta-analysis conducted by the MiBioGen Consortium (*n* = 13,266). Appendicular lean mass data were obtained from the UK-Biobank (*n* = 450,243). We conducted bidirectional two-sample Mendelian randomization (MR) analysis using summary-level data from GWAS to investigate the causal relationship between *Lachnospiraceae* and ALM. Additionally, we employed a drug-targeted MR approach to assess the causal relationship between AOPEP and ALM. Finally, a two-step MR was employed to quantitatively estimate the proportion of the effect of *Lachnospiraceae* on ALM that is mediated by AOPEP. Cochran's *Q* statistic was used to quantify heterogeneity among instrumental variable estimates.

**Results:**

In the MR analysis, it was found that an increase in genetically predicted *Lachnospiraceae* [OR = 1.031, 95% CI (1.011–1.051), *P* = 0.002] is associated with an increase in ALM. There is no strong evidence to suggest that genetically predicted ALM has an impact on *Lachnospiraceae* genus [OR = 1.437, 95% CI (0.785–2.269), *P* = 0.239]. The proportion of genetically predicted *Lachnospiraceae* mediated by AOPEP was 34.2% [95% CI (1.3%−67.1%)].

**Conclusion:**

Our research reveals that increasing *Lachnospiraceae* abundance in the gut can directly enhance limb muscle mass and concurrently suppress AOPEP, consequently mitigating limb muscle loss. This supports the potential therapeutic modulation of gut microbiota for sarcopenia. Interventions such as drug treatments or microbiota transplantation, aimed at elevating *Lachnospiraceae* abundance and AOPEP inhibition, synergistically improve sarcopenia in the elderly, thereby enhancing the overall quality of life for older individuals.

## 1 Introduction

Sarcopenia is a progressive, systemic condition characterized by the pathological deterioration of skeletal muscle strength, quantity, and quality, and it is frequently observed in the elderly population (Zhang et al., 2023). In individuals aged 60 and above, the prevalence of muscle loss is estimated to be between 10 and 27%. Predictions indicate that by the year 2050, approximately two billion people globally will be affected by muscle loss (Petermann-Rocha et al., 2022). Muscle loss is associated with an increased risk of various adverse conditions, including limited mobility, heightened susceptibility to illnesses, increased hospitalization, and elevated mortality rates (Cruz-Jentoft et al., 2010). However, effective treatment methods are currently lacking (Cohen et al., 2015). Therefore, it is essential to explore economically effective treatment methods or provide a basis for therapeutic directions.

Recently, the concept of the “gut-muscle” axis regulation has been proposed and three specific gut microbiota species (Grahnemo et al., 2023) have been identified to be closely associated with appendicular lean mass (Bäckhed et al., 2007). In mice, it has been established that the gut microbiota can modulate muscle mass (Janssen et al., 2004; Kim et al., 2017), and similar findings have been corroborated in humans as well (Lv et al., 2021). Research conducted by the Hunt study queue has demonstrated an association between *Coprococcus come, Dorea longicatena*, and *Eubacterium ventriosum* with higher ALM (Grahnemo et al., 2023), but the mechanism is unclear. The AOPEP play a pivotal role in preserving skeletal muscle mass and myodystony (Schmidt et al., 2009; Hsu et al., 2021). Changes in the abundance of *Lachnospiraceae* are commonly associated with sarcopenia and disorders of muscle tone (Sampson et al., 2016; Picca et al., 2019; Ticinesi et al., 2020; Štorkánová et al., 2021). Consequently, AOPEP might be a potential mediator between *Lachnospiraceae* and ALM.

However, the results regarding the *Lachnospiraceae* and its relation to limb muscle mass have been controversial to date (Vacca et al., 2020). Observational studies have indicated that in elderly individuals with sarcopenia, the abundance of the *Lachnospiraceae* is significantly lower compared to control groups (Picca et al., 2019; Ticinesi et al., 2020; Štorkánová et al., 2021). Conversely, other studies have shown that within the *Firmicutes phylum*, the *Lachnospiraceae* is positively correlated with body fat and waist circumference, while being negatively associated with muscle mass and physical activity levels (Palmas et al., 2021). These discrepancies may arise from limited sample sizes, study design limitations, and confounding factors beyond the scope of existing research.

Mendelian randomization (MR) (Beeghly-Fadiel et al., 2020; Titova et al., 2020; Ahmed et al., 2021; Lu et al., 2021) represents a promising causal inference approach employing genetic variation as an instrumental variable to ascertain the impact of exposure factors on outcomes within observational datasets. This method has the capacity to mitigate the influence of non-measurement errors and confounding variables, all while circumventing issues of reverse causality by leveraging the principles of Mendelian inheritance. Our primary objectives encompass (i) the investigation of a potential causal relationship between *Lachnospiraceae* and ALM and (ii) the evaluation of the degree to which AOPEP mediates the effects of *Lachnospiraceae* on ALM.

## 2 Materials and methods

### 2.1 Study design

**Figure 1:**
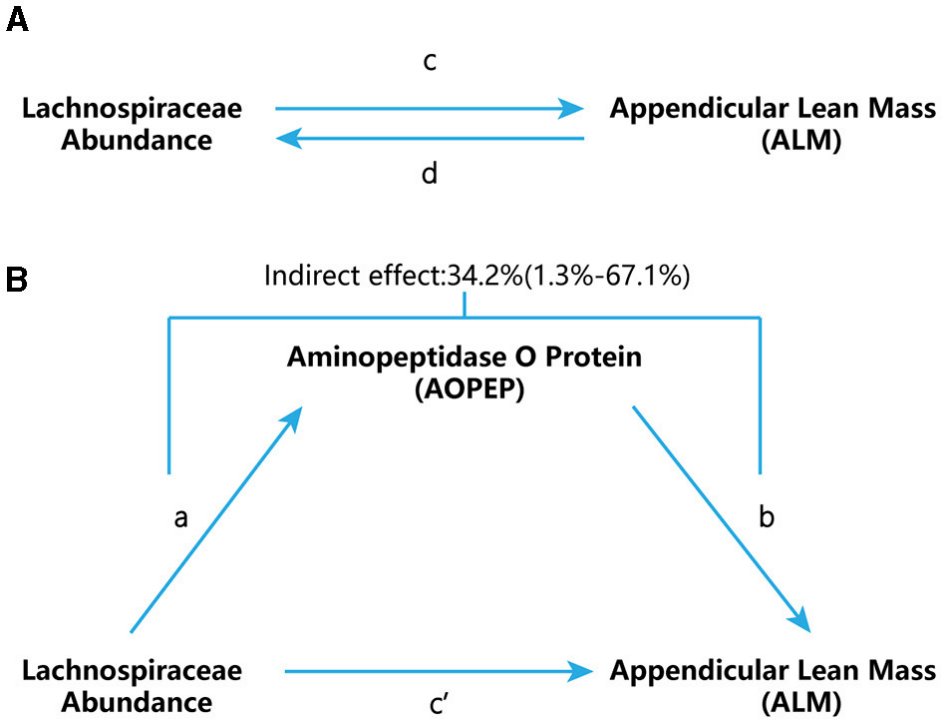
Study flow chart. **(A)** “c” represents the total effect when genetically predicted Lachnospiraceae serves as the exposure variable and ALM as the outcome. “d” signifies the total effect when genetically predicted ALM is the exposure variable and Lachnospiraceae is the outcome. **(B)** (i) Indirect effect, which was assessed using a two-step approach that involves “a” as the total effect of Lachnospiraceae on AOPEP and “b” as the effect of AOPEP on ALM. (ii) Direct effect (c′= c – a × b).

In this research, we conducted a two-sample MR study utilizing summary data from GWAS datasets to assess the relationship among gut microbiota, AOPEP expression and ALM in Figure 1. We also performed sensitivity analyses to validate the reliability of our findings. MR hinges upon three fundamental assumptions: (1) the instrumental variable must exhibit a strong association with the exposure factor; (2) the instrumental variable should not be correlated with any confounding factors associated with the “exposure-outcome” relationship; (3) the instrumental variable should only influence the outcome variable through the exposure factor. These assumptions are integral to the validity of MR and are rigorously tested throughout our study (Bandres-Ciga et al., 2020; Chen et al., 2020; Feng et al., 2020; Jones et al., 2020; Larsson et al., 2020; Saunders et al., 2020; Scheller Madrid et al., 2020; Zhu et al., 2020).

### 2.2 Data source

The genetic variation data for gut microbiota were derived from the Meta-analysis conducted by the MiBioGen Consortium, representing the most extensive investigation to date into the genomic scope of gut microbiota composition (Kurilshikov et al., 2021). This expansive study encompassed a cohort of 18,340 individuals drawn from 24 distinct cohorts, with the majority being of European descent (*n* = 13,266). In the MiBioGen Consortium research, the genus was the lowest taxonomic level. A total of 131 genera were identified, each with an average abundance exceeding 1%, including 12 unknown genera (Li et al., 2022). GWAS data concerning ALM were procured from the UK Biobank (https://www.ukbiobank.ac.uk/), with access facilitated through the IEU_Gwas platform. This dataset encompassed 450,243 samples and comprised a staggering 18,071,518 SNP loci, furnishing a robust foundation for our investigation (Pei et al., 2020).

### 2.3 Instrumental variables

In order to maximize the utility of instrumental variables (IVs), the following selection criteria were employed: (1) SNP significance threshold: SNPs within loci demonstrating a significant threshold (*P* < 1.0 × 10^−5^) with each genus were considered as potential instrumental variables. (2) Reference panel utilization: The 1000 genomes project European sample data served as the reference panel for calculating linkage disequilibrium (LD) between SNPs. Among SNPs with an LD coefficient (*R*^2^) <0.01 within a 30 kb window, only those with the lowest *P*-values and an *F*-statistic >10 were retained. (3) Handling of Palindromic SNPs: in cases involving palindromic SNPs, allelic frequency information was used to infer the forward strand alleles (Sanna et al., 2019; Li et al., 2022). In this study, only cis-eQTLs (expression quantitative trait loci) in the trans configuration were considered for generating genetic instruments. These were defined as eQTLs located within 1 Mb on either side of the target gene. To maximize the instrumental strength for each genus, SNPs used as instruments were allowed to exhibit low linkage disequilibrium with each other (*R*^2^ <0.30) (Willer et al., 2013; Huang et al., 2021).

### 2.4 Statistical analysis

#### 2.4.1 Primary analysis

Figure 1 provides a schematic overview of our analysis. We conducted a bidirectional two-sample MR to assess the reciprocal causation between *Lachnospiraceae* and ALM (Figure 1A), representing the total effect. To estimate MR effects, we employed various methods to ensure robustness. The Inverse Variance Weighting (IVW) method, which combines Wald ratios of causal effects for each SNP through meta-analysis, was utilized as the primary approach. In addition to IVW, we complemented our analysis with the MR-Egger and weighted-median methods, each catering to different assumptions of instrument validity. The IVW method relies on the assumption that all SNPs are valid instrumental variables, enabling accurate estimation results. MR-Egger, on the other hand, assesses directional pleiotropy of instrumental variables, with the intercept offering an estimate of the average pleiotropy of genetic variation. The weighted median method boasts higher precision, indicated by a smaller standard deviation, when compared to MR-Egger. Importantly, the weighted median method provides consistent estimates even in the presence of horizontal pleiotropy, even if up to 50% of the genetic variants are deemed invalid instruments.

#### 2.4.2 Mediation analysis

**Figure 2:**
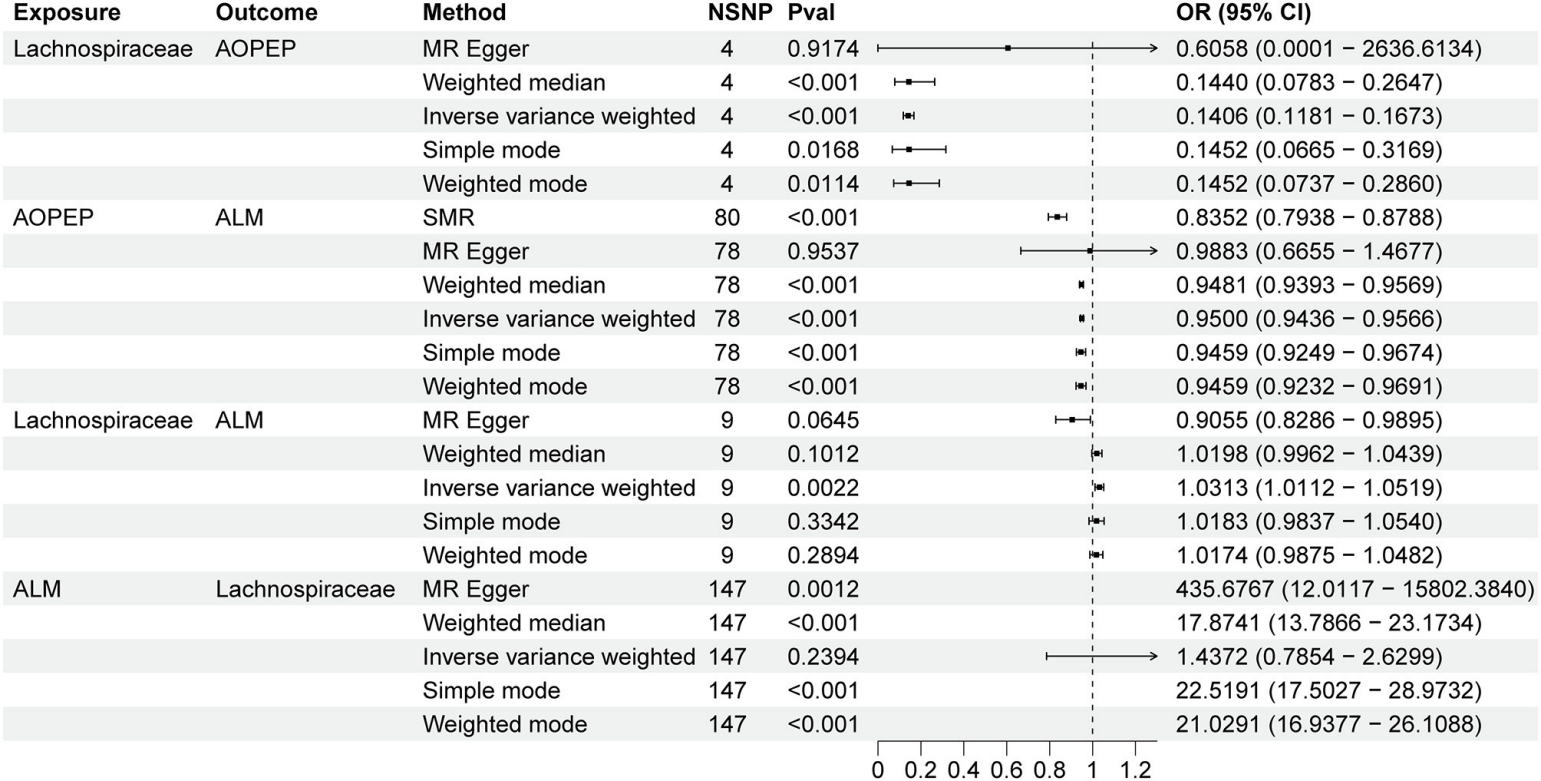
Forest plot to visualize the causal effects of AOPEP with *Lachnospiraceae* and ALM.

We further employed a two-step MR design for conducting a mediation analysis to explore whether AOPEP mediates the causal pathway from *Lachnospiraceae* to the ALM outcome (Figure 1B). We took examples from drug target MR analysis to investigate the effect of AOPEP on ALM. The overall effect can be decomposed into indirect effects (mediated through the mediator) and direct effects (effects without mediation). The total impact of *Lachnospiraceae* on ALM can be separated into (1) the direct effect of *Lachnospiraceae* on ALM (c′ in Figure 1B) and (2) the indirect effect of *Lachnospiraceae* on ALM mediated through AOPEP (a × b in Figure 1B). We calculated the percentage mediated by the mediation effect by dividing the indirect effect by the total effect, simultaneously computing the 95% confidence interval.

This study employed the Summary Data-based Mendelian Randomization (SMR) method, utilizing eQTLs as instrumental variables to generate effect estimates (Zhu et al., 2016). This approach investigates associations between gene expression levels and the outcomes of interest, utilizing summary-level data from GWAS and eQTL studies. SMR software version 1.3.1 was used for allele harmonization and analysis. The IVW method was primarily employed for effect estimation. Allele harmonization and analysis were conducted using the TwoSampleMR package in R software version 4.3.0. All statistical tests were two-tailed, with statistical significance defined as *P* < 0.05.

## 3 Results

### 3.1 The association between *Lachnospiraceae* and AOPEP

**Figure 3:**
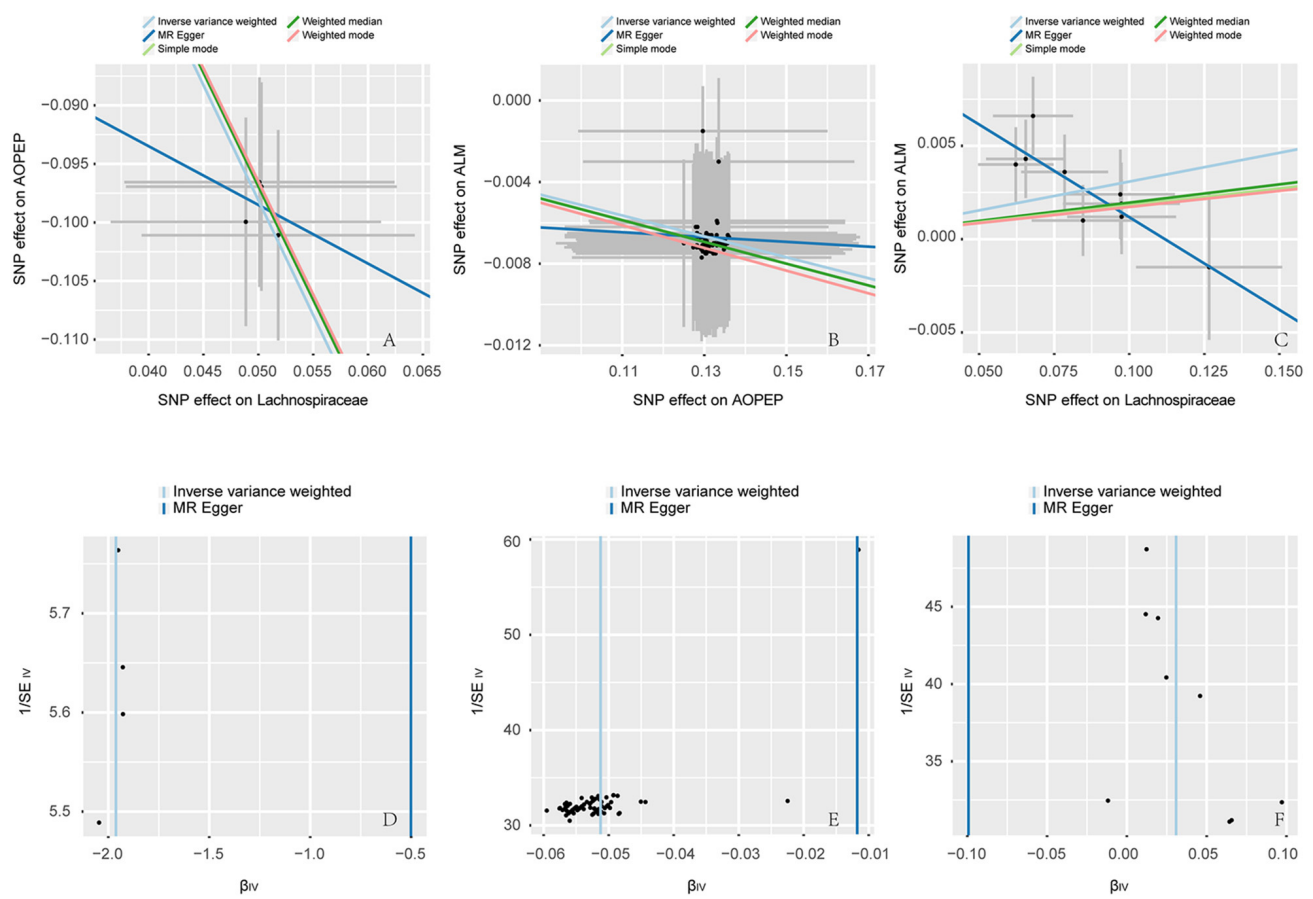
Scatter and funnel plot. **(A–C)** are scatter plots, the horizontal axis represents the SNP effect on exposure, while the vertical axis illustrates the SNP effect on the outcome. The IVW method represents the causal relationship between exposure and outcome through the slope of the line. A positive slope indicates a positive correlation, while a negative slope suggests a negative correlation. Each point in **(A–C)** represents a SNP used for MR analysis, with the length of the line segment indicating the standard error. **(D–F)** are funnel plots employed to assess heterogeneity in SNP. The horizontal axis representing the effect of SNP and the vertical axis representing precision.

Incorporating relevant SNPs associated with the gut microbiota of the *Lachnospiraceae* from MiBioGen and cis-eQTLs for AOPEP gene expression from eQTLGen resulted in a total of 1,197 and 722 SNPs, respectively. Out of these, four instrumental variables suitable for MR analysis were identified, each with an F-statistic exceeding 10, indicating a strong association with the exposure factor. This rigorous selection process minimized the potential bias introduced by weak instrumental variables. Based on predictive outcomes, a close relationship between gut microbiota and AOPEP gene expression was revealed. The IVW method estimated an effect size with an OR of 0.141 [95% CI (0.118–0.167), *P* = 5.879 × 10^−108^] in Figure 2. The Weighted Median method also demonstrated a similar causal relationship with an OR of 0.144 [95% CI (0.007–0.264), *P* = 4.482 × 10^−10^], while Simple Mode and Weighted Mode yielded results consistent with the aforementioned methods. However, the Mr-Egger method did not yield comparable findings in Figure 3A. To assess the stability of these results, additional Mr-Egger and Mr-PRESSO tests were conducted on the included SNP loci. No potential horizontal pleiotropy (*P* > 0.05) was detected in either test, and funnel plots (Figure 3D) revealed no evidence of bias in the study. Corrected Cochran's Q statistics indicated no significant heterogeneity in the effects of the included SNPs (*P* > 0.90). Furthermore, leave-one-out sensitivity analyses were employed to evaluate the influence of each SNP locus on the overall causal relationship. The results demonstrated no significant differences in the observed causal relationship when systematically removing individual SNPs and reanalyzing, underscoring that the estimated effect could not be attributed to any single genetic instrument.

### 3.2 The association between AOPEP and ALM

A total of 722 cis-eQTLs pertaining to the AOPEP gene were identified from the eQTLGen, with 78 of these exhibiting significant associations. SMR analysis results unveiled a significant correlation between increased AOPEP gene expression and decreased ALM [OR = 0.835, 95% CI (0.793–0.878), *P* = 3.81 × 10^−12^]. This suggests that suppressing AOPEP gene expression may lead to an increase in ALM. The same causal relationship was also demonstrated in the IVW-MR analysis [OR = 0.95, 95% CI (0.943–0.956), *P* = 1.04 × 10^−48^] as shown in Figures 2, 3B. The other MR methods results and the results of SMR are shown in Supplementary material.

### 3.3 The association between *Lachnospiraceae* and ALM

A total of 1,197 SNPs related to the *Lachnospiraceae* from MiBioGen and 1,193 SNPs associated with limb muscle mass from GWAS studies were incorporated for investigation. Among these, nine SNPs were selected for MR analysis. All SNPs exhibited *F*-statistics exceeding 10, signifying strong associations with the exposure factor. This rigorous selection process minimized potential bias introduced by weak instrumental variables. Based on IVW-MR predictions, there was causal association between the *Lachnospiraceae* and ALM [OR = 1.031, 95% CI (1.011–1.051), *P* = 0.002]. The other methods did not provide any indication of a causal relationship from *Lachnospiraceae* to ALM in Figure 3C. In the reverse MR analysis, the IVW method [OR = 1.437, 95% CI (0.785–2.269), *P* = 0.239] indicates that there is no causal relationship from ALM to *Lachnospiraceae*. Considering the IVW as the primary effect estimation indicator, it can be concluded that there is only causal relationship between the *Lachnospiraceae* and ALM. The proportion of genetically predicted *Lachnospiraceae* mediated by AOPEP was 34.2% [95% CI (1.3%−67.1%)].

**Figure 4:**
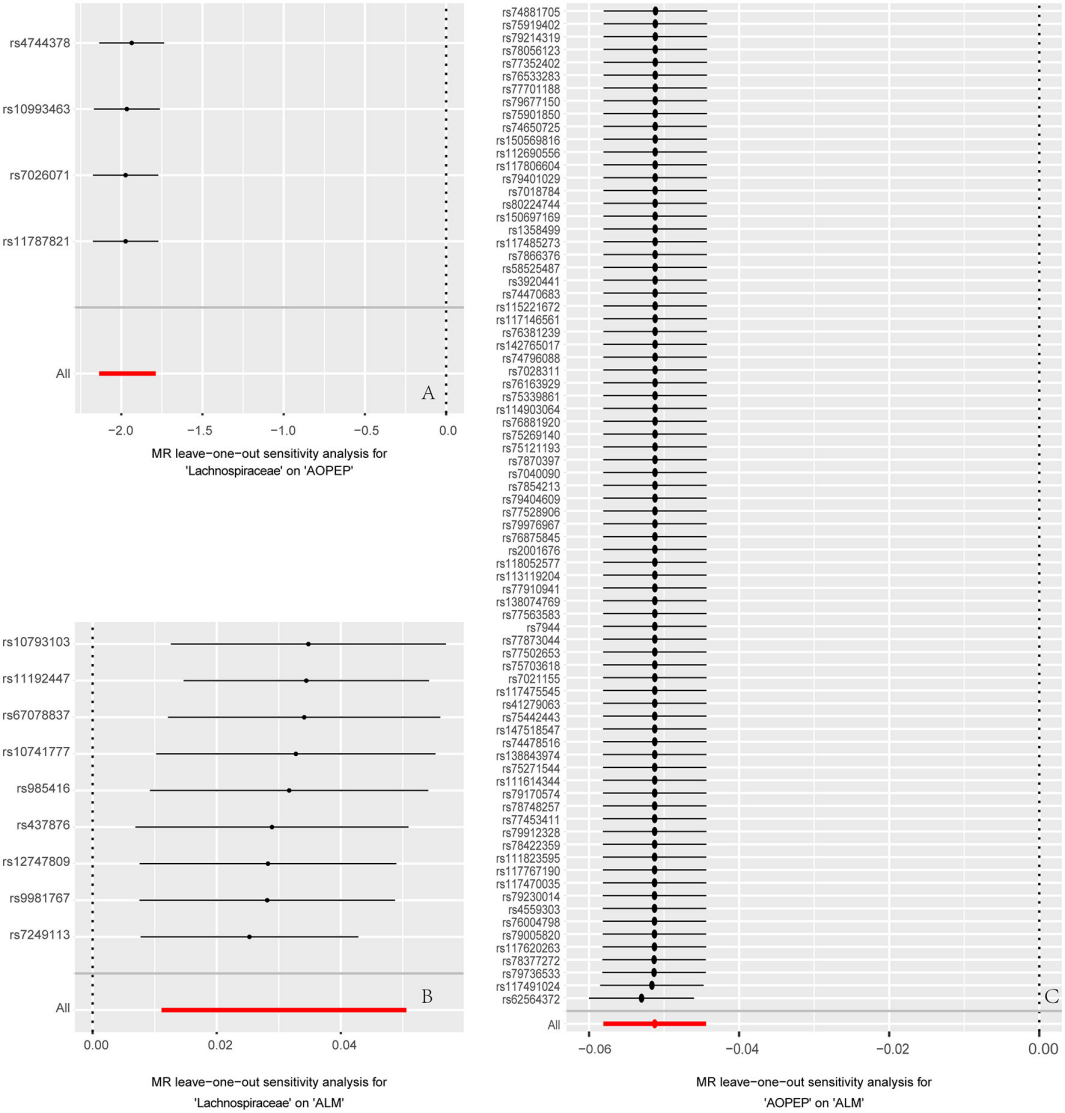
Forest plot to visualize the impact of removing a single SNP on the overall effect. **(A)** Represents the MR analysis between *Lachnospiraceae* and AOPEP. **(B)** Represents the MR analysis between *Lachnospiraceae* and ALM. **(C)** Represents the MR analysis between AOPEP and ALM. The horizontal axis represents beta values, while the vertical axis depicts the SNP ID and the cumulative effect size after the removal of individual SNP.

**Figure 5:**
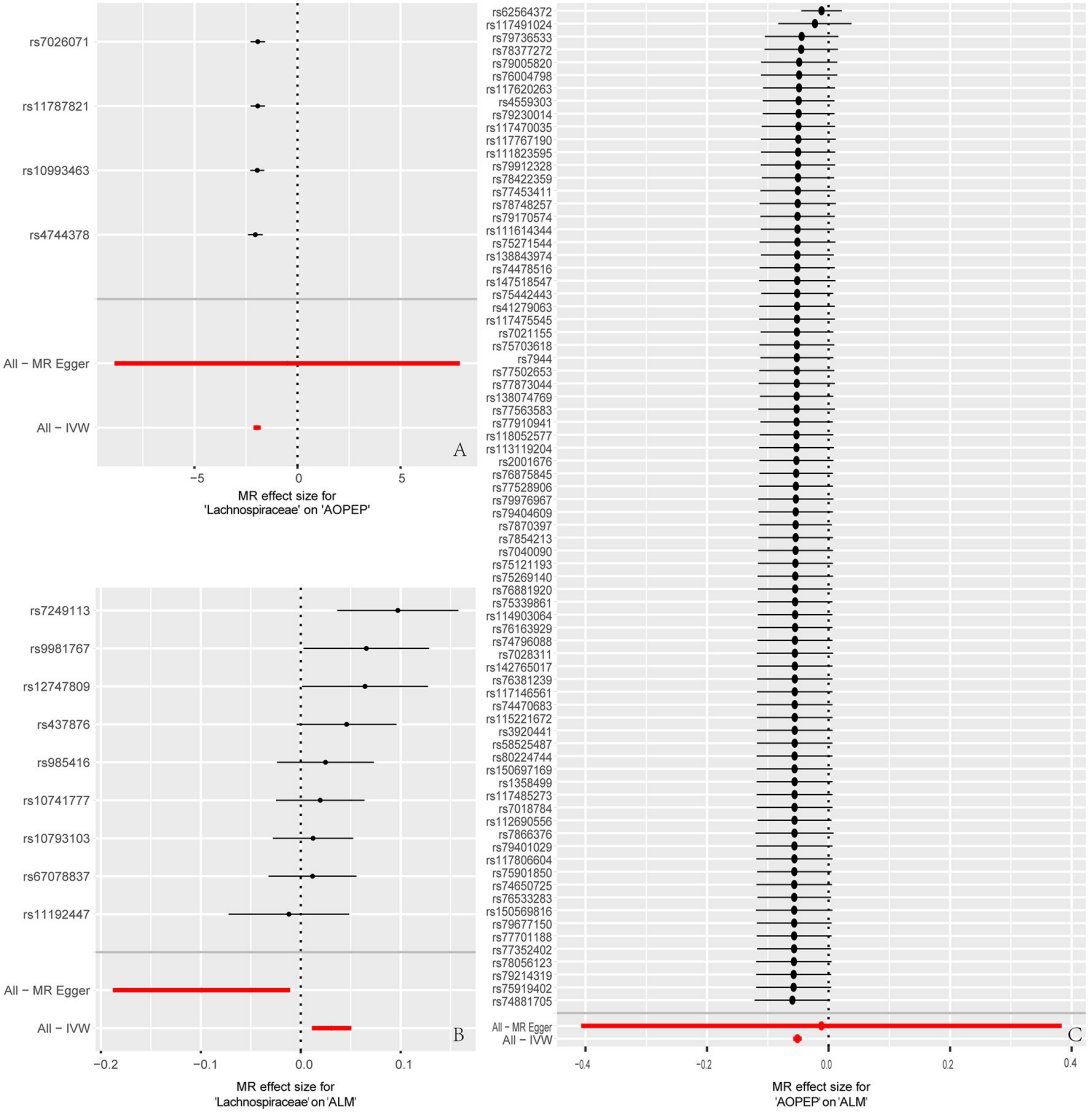
Forest plot to visualize causal effects of each single SNP on outcome risk. **(A)** Represents the individual effects of each single SNP on AOPEP expression in the MR analysis comparing *Lachnospiraceae* and AOPEP. **(B)** Represents the individual effects of each single SNP on ALM risk in the MR analysis comparing *Lachnospiraceae* and ALM. **(C)** Represents the individual effects of each single SNP on ALM risk in the MR analysis comparing AOPEP and ALM. The horizontal axis corresponds to beta values, and the vertical axis displays SNP ID along with the overall effect size beta values obtained through both MR-Egger and IVW methods.

At last, various sensitivity analyses were employed to examine and rectify the presence of pleiotropy in causal estimates in Figure 4. IVW and MR-Egger were used to estimate the causal relationship between genetically predicted among *Lachnospiraceae*, AOPEP and ALM in Figure 5. Cochran's Q test and funnel plots (Figures 3D–F) indicated that there was no evidence of heterogeneity and horizontal pleiotropy among these SNPs in the causal relationships (Supplementary Tables 2, 4, 6, 8, 10, 12).

## 4 Discussion

While recent research has validated the relationship between *Lachnospiraceae* and ALM, the evidence has been limited to observational studies, which could be influenced by confounding factors. Our study aimed to establish the causal effect of *Lachnospiraceae* on ALM. We utilized MR analysis to investigate the association between *Lachnospiraceae* and ALM based on existing GWAS data and to demonstrate whether their relationship is mediated through AOPEP. Our results indicate a genetically predicted increased risk in ALM associated with *Lachnospiraceae* (a 1 SD increase in *Lachnospiraceae* is linked to a 3.1% increase in ALM risk), with 34.2% of this effect being mediated by AOPEP.

To our knowledge, this is the first time that MR methods have been employed to study the causal relationship between *Lachnospiraceae* and ALM, and it confirms Aminopeptidase O Protein as the mediator. Our findings are consistent with results from observational studies. Nikkhah et al. (2023) showed through a meta-analysis of the results of seven human studies and five animal studies that the genera that decreased in individuals with age-related sarcopenia were *Lachnospiraceae, Fusicatenibacter, Roseburia, Eubacterium, Lachnoclostridium*, and *Slackia*. Observational studies have shown that *Lachnospiraceae* and Aminopeptidase O Protein co-regulate angiotensin and muscle metabolism (Bai et al., 2021; Bajaj et al., 2021; Schulz et al., 2021; Sun et al., 2021). Additionally, research has identified pathogenic genes related to muscle atrophy, such as AOPEP (Fevga et al., 2022; Garavaglia et al., 2022; Menden et al., 2022; Zech et al., 2022; Lin et al., 2023; Thomsen et al., 2023), suggesting a potential novel approach for patients with cachexia to improve muscle symptoms by altering the composition of gut microbiota. Both investigations adopted an observational design. Firstly, they exhibited relatively low response rates across the two groups. Secondly, the outcomes were susceptible to greater influence from reverse causality and other potential confounding effects compared to MR analyses.

The human gastrointestinal tract harbors a vast and intricate microbial community known as the gut microbiota, comprising as many as thousands of bacterial species in adults (Matijašić et al., 2020). Notably, the *phyla Firmicutes* and *Bacteroidetes* collectively dominate about 95%, exerting substantial influence over the overall gut microbiota's functionality (Deng and Swanson, 2015). This intricate symbiotic relationship between the gut microbiota and the human host has evolved over millennia, with the microbiota playing a pivotal role in various aspects of human health. The gut microbiota and its metabolic byproducts contribute significantly to functions such as aiding in food digestion and nutrient absorption, synthesizing vitamins and energy, safeguarding the integrity of the intestinal mucosal barrier, and playing essential roles in crucial metabolic processes, immune regulation (Brusca et al., 2014; Sathyabama et al., 2014), and defense against pathogenic invaders. A plethora of studies has linked alterations in the composition of the gut microbiota to the development of numerous chronic diseases, including inflammatory bowel disease (Koboziev et al., 2014), metabolic disorders (Fukuda and Ohno, 2014), obesity, malnutrition, neurodegenerative diseases (de Theije et al., 2014), cardiovascular disorders (Vinjé et al., 2014), and muscle metabolism (Schmidt et al., 2009; Hsu et al., 2021).

Skeletal muscle is the largest organ in the human body, accounting for ~40% of body mass (Guridi et al., 2015). Furthermore, skeletal muscle serves various other functions, including acting as a reservoir for major macronutrients, protecting internal organs, regulating core temperature, and communicating with other organs within the body through the release of cytokines and growth factors (Pedersen and Febbraio, 2008). The muscles of the limbs constitute 75% of the total body muscle mass (Heymsfield et al., 1990; Chaston et al., 2007). Therefore, muscular dystrophy is largely manifested as a reduction in muscle mass in the limbs. Existing research has demonstrated the correlation between ALM and physical activity, bone density, and metabolic function. With advancing age, there is a reduction in skeletal muscle mass, accompanied by an increase in fat infiltration and muscle fibrosis. Over time, this phenomenon can impact limb functionality and lead to paralysis (Nikkhah et al., 2023). Therefore, preserving muscle mass holds significant importance.

Systemic inflammation and resistance to metabolite synthesis play a critical pathophysiological role in muscle atrophy (Holeček, 2017). Elevated levels of muscle TNF-α, NF-κB, and IL-6 can induce muscle atrophy by activating muscle atrophy-related and protein-hydrolyzing genes (MAFbx and MurF1). *Lachnospiraceae* has the ability to synthesize short-chain fatty acids (SCFAs) (Ticinesi et al., 2020; Štorkánová et al., 2021). Butyrate can increase the content of tight junction proteins and plaque proteins involved in cell-to-cell connections (Anderson and Van Itallie, 2009), thereby contributing to the enhancement of intestinal barrier function and reducing endotoxin entry into the bloodstream. Additionally, butyrate can bind and activate the nuclear transcription factor PPARγ, counteracting the NF-κB signaling pathway, thus alleviating inflammation and preventing muscle loss (Alex et al., 2013). After muscle tissue intake of acetate increases, the catalytic activity of acetyl-CoA synthetase is enhanced, leading to the production of a large amount of acetyl-CoA and an increase in cytoplasmic AMP, resulting in an elevated AMP/ATP ratio (Itsuki-Yoneda et al., 2007). This decrease in glycolysis reduces and increases the storage of glycogen in skeletal muscles (Fushimi et al., 2001). Furthermore, the increased AMP/ATP ratio leads to increased AMPK phosphorylation, upregulation of lipolysis genes LCACD, 3 KACT, and PPAR, thereby reducing fat infiltration in the muscles (Yamashita et al., 2009). The above mechanistic studies illustrate that *Lachnospiraceae* can enhance limb muscle mass, consistent with our findings.

Aminopeptidase O Protein (AOPEP) is a protein-coding gene. This gene encodes a member of the M1 zinc aminopeptidase family. The encoded protein is a zinc-dependent metallopeptidase that catalyzes the removal of amino acids from the N-terminus of proteins or peptides. This protein plays a role in the generation of angiotensin in the renin-angiotensin system, and its associated pathways include peptide hormone metabolism and muscle protein metabolism (Wu et al., 2011). Mutations in AOPEP were found to be associated with muscle atrophy and impaired muscle tone in a large-scale multicenter study (Fevga et al., 2022; Garavaglia et al., 2022; Zech et al., 2022; Lin et al., 2023). However, the mechanism by which AOPEP acts on ALM is currently unclear. *Lachnospiraceae* is involved in angiotensin and AOPEP metabolism (Sun et al., 2021). Therefore, AOPEP may lead to limb muscle loss by mediating *Lachnospiraceae* role in muscle metabolism. The above mechanisms, both directly and indirectly, elucidate the relationship between Lachnospiraceae, AOPEP, and ALM, providing support for our research findings.

Transplanting gut microbiota to increase the abundance of *Lachnospiraceae* in patients has been shown to improve the production of short-chain fatty acids (SCFAs) in the gut. This modulation helps regulate inflammation and the systemic immune environment, thereby alleviating hepatic encephalopathy caused by cirrhosis (Bajaj et al., 2019) and severe acute malnutrition (Castro-Mejía et al., 2020). As mentioned earlier, inflammation plays a crucial role in muscular dystrophy. Therefore, modulating the gut microbiota to enhance *Lachnospiraceae* abundance holds great potential for treating muscular dystrophy. Our study provides a basis for increasing *Lachnospiraceae* abundance in the gut as a therapeutic approach for muscular dystrophy, further enriching the understanding of the role of microbiota in disease treatment. Additionally, AOPEP, as an intermediary factor, offers new clues for exploring the pathophysiological mechanisms and drug development. Furthermore, since muscular dystrophy is a risk factor for cognitive impairment, treating muscular dystrophy may contribute to reducing cognitive decline in the elderly (Shimada et al., 2021; Du et al., 2022). Similar research approaches can be employed to explore more intermediary factors, providing robust evidence for investigating the pathophysiological mechanisms and metabolic pathways of diseases.

However, there are still some limitations in our research. Firstly, our analysis was conducted using a European population, which limited its generalizability to other populations. Secondly, the number of cases in the ALM GWAS dataset was relatively small, and it was hoped that larger GWAS datasets will be available for future validation. Thirdly, even though we took measures to identify and eliminate outliers and variations, we cannot entirely rule out the possibility of horizontal pleiotropy affecting our results. Fourth, our study utilized summary-level statistics rather than individual-level data, which prevented us from further exploring causality between subgroups, such as females and males. Fifth, our research indicated a genetic prediction rate of 34.2% for muscle loss mediated by AOPEP, which was relatively low. Considering the involvement of other factors like vitamins, exercise, fatty acids, and immunoregulatory peptides in muscle metabolism, further research is needed to quantify the contributions of these other mediators. Finally, further *in vivo* and *in vitro* experiments are needed to validate the role of AOPEP in the ALM.

## 5 Conclusion

Our study, utilizing MR analysis, demonstrates that increasing the abundance of *Lachnospiraceae* in the gut can directly enhance limb muscle mass and also suppress AOPEP, thereby indirectly reducing limb muscle loss. This provides evidence for the modulation of the gut microbiota as a therapeutic approach for sarcopenia. Therefore, interventions such as drug treatments or microbiota transplantation aimed at augmenting the abundance of *Lachnospiraceae* and AOPEP inhibitor synergistically improve sarcopenia in the elderly, thereby enhancing the overall quality of life for older individuals.

## Data availability statement

The datasets presented in this study can be found in online repositories. The names of the repository/repositories and accession number(s) can be found in the article/Supplementary material.

## Ethics statement

Ethical approval was not required for the studies involving humans because this two-sample MR study is based on publicly available summary data from genome-wide association studies (GWAS) and expression quantitative trait loci (eQTL) studies. All of these studies have obtained approval from the relevant institutional review boards, and participants have provided informed consent. The studies were conducted in accordance with the local legislation and institutional requirements. Written informed consent for participation was not required from the participants or the participants' legal guardians/next of kin in accordance with the national legislation and institutional requirements because this two-sample MR study is based on publicly available summary data from genome-wide association studies (GWAS) and expression quantitative trait loci (eQTL) studies. All of these studies have obtained approval from the relevant institutional review boards, and participants have provided informed consent. The manuscript presents research on animals that do not require ethical approval for their study.

## Author contributions

BG: Conceptualization, Investigation, Methodology, Visualization, Writing – original draft, Writing – review & editing. ZZ: Data curation, Visualization, Writing – review & editing. JC: Investigation, Software, Supervision, Writing – review & editing. SZ: Formal analysis, Project administration, Validation, Writing – review & editing. SJ: Visualization, Writing – original draft. WY: Conceptualization, Formal analysis, Supervision, Writing – review & editing. YL: Formal analysis, Visualization, Writing – review & editing. KW: Investigation, Software, Validation, Writing – review & editing. JL: Data curation, Investigation, Validation, Writing – review & editing. YZ: Conceptualization, Data curation, Formal analysis, Investigation, Supervision, Writing – review & editing.
